# Management of a Ruptured Pseudoaneurysm of Common Hepatic Artery Following Pancreaticoduodenectomy

**DOI:** 10.1100/tsw.2007.249

**Published:** 2007-10-12

**Authors:** M. Abu-Hilal, M. J. Hallam, B. A. Zeidan, N. W. Pearce

**Affiliations:** Hepato Pancreatico Biliary Surgery Unit, Southampton General Hospital, Tremona Road, Southampton, SO16 6YD, UK

**Keywords:** Pancreaticoduodenectomy, pseudoaneurysm, pancreatic fistula, pancreatic leak, haemostatic products, haemorrhage

## Abstract

Postoperative pseudoaneurysm formation is one of the most feared complications of pancreatic leak following pancreaticoduodenectomy (PD). Surgical repair may be compromised due to a persistent enzymatic insult on the repaired vessel; therefore, preventive measures should be adopted. We report a case of ruptured hepatic artery pseudoaneurysm occurring 12 days following PD in a patient with a postoperative pancreatic fistula. Emergency surgery revealed that the pseudoaneurysm was situated at the point of surgical transfixation of the gastroduodenal artery. The pseudoaneurysm was successfully managed by under-running of the bleeding point combined with the direct application of hemostatic products to the bleeding surface (TachoSil and Tisseel to act as a barrier from the pancreatic secretions.

## INTRODUCTION

Outcomes after pancreaticoduodenectomy (PD) (Whipple's procedure) have improved significantly in recent years, with reported operative mortality rates of less than 5%[1]. However, complications still occur in approximately 25% of patients[2]. The most common cause of significant morbidity following PD is leakage at the site of pancreatic anastomosis that often results in a peripancreatic fluid collection, abscess, or the development of pancreatic fistula[1]. The major risk factors for leak appear to be the texture of the pancreas and the size of the pancreatic duct[1]. Pancreatic fistulae are not only potentially life threatening, but also can lead to prolonged hospital stays, which in turn, can have a significant impact on quality of life and treatment costs[2,3]. The most serious complication of pancreatic fistulae is the formation of an arterial pseudoaneurysm, which may lead to rupture and hemorrhage, usually after the 5$^{th}$ postoperative day[4]. We report a rare case of a ruptured hepatic artery pseudoaneurysm occurring 12 days following PD. The pseudoaneurysm was successfully treated with a combination of surgical suture and the application of a fibrinogen and thrombin-coated collagen sponge (TachoSil™), and a layer of fibrin glue (Tisseel™).

## CASE REPORT

An 83-year-old man previously in good health underwent a PD for distal common bile duct cholangiocarcinoma in June 2005. During surgery, the patient lost 900 ml of blood, but did not require an intraoperative transfusion and the operation was otherwise uneventful. On the 7th day postoperatively, amylase concentration in the drainage fluid was found to be elevated (>1000 i.u./l). Daily drain output was 75 ml. He was diagnosed with a small-volume pancreatic leak; however, following conservative management, the amount of drainage fluid decreased over the next 5 days.

On the 12th day postoperatively, one liter of fresh blood discharged rapidly into the patient's intra-abdominal drain and he felt generally unwell. He underwent immediate emergency surgery, where it was revealed that he had a partially contained pancreatic leak with an associated ruptured inflammatory pseudoaneurysm of the common hepatic artery at the site of surgical transfixation of the gastroduodenal artery. The rupture was repaired with 4-0 Prolene suture, the repaired artery was then wrapped with a piece of TachoSil™, and a thick layer of the fibrin glue (Tisseel™ 5 ml) was applied topically as a preventative measure. Additionally, a 20 French drainage tube was placed precisely at the anastomotic site to control the pancreatic leak. The patient was transferred to the intensive care unit where he was monitored, and eventually he was discharged on the 29th postoperative day. Postoperative Doppler ultrasound confirmed normal hepatic artery flow. The patient remains well 1 year later.

## DISCUSSION

PD is the gold-standard treatment for patients with periampullary lesions. Advances in anesthetics, improvements of surgical techniques and materials, and postoperative care, as well as the management of postoperative complications, have helped to improve survival, with some tertiary centers reporting mortality rates approaching 1%[5]. Despite a dramatic improvement in the mortality rate, the postoperative morbidity remains high[4], although this varies from center to center.

Many postoperative complications respond to nonsurgical treatment[6]; however, hemorrhagic complications continue to cause challenges in pancreatic surgery, since bleeding can be difficult and time consuming to bring under control[7]. Rumstadt et al. emphasized that sentinel bleeding in patients with a pancreatic leak is frequently secondary to a ruptured pseudoaneurysm and should be taken very seriously, prompting immediate laparotomy or angiography[8]. Different studies have emphasized the difficult management associated with arterial hemorrhage secondary to pancreatic anastomotic leak following PD, and have suggested a completion pancreatectomy and a new pancreaticojejunostomy in some cases[9].

The difficulty in managing such a situation is a result of the generalized damage caused to the arterial wall by activated pancreatic enzymes. Surgical suture alone is often insufficient to repair the site of a pseudoaneurysm without completely occluding the vessel and, in a previous similar case, we observed a breakdown of the vascular repair, presumably due to the proximity of the pseudoaneurysm to the site of pancreatic leak. The use of additional hemostatic materials, such as TachoSil™ and Tisseel™, aims to create a protective barrier against pancreatic secretions, thus providing effective hemostasis and reducing the need for additional sutures that may obliterate the lumen of the vessel.

Physiologically, on contact with body fluids, the procoagulant substances contained in TachoSil™ form a solid, mechanically stable fibrin clot that firmly joins the collagen fleece with the wound surface and can provide a valid protective layer[7].

Lymphoadenectomy is a routine part of our PD that may leave the skeletonized vessel in direct contact with pancreatic secretions in cases of pancreatic leak, thus increasing the risk of pseudoaneurysm formation and subsequent hemorrhaging. This interesting case was important to us not only for the management of bleeding pseudoaneurysms, but also in terms of changing our elective practice. We now use these hemostatic products as a preventive measure in patients with high risk of pancreatic leak (small duct, soft pancreas). This is achieved simply by applying the hemostatic product directly to the skeletonized vessels' dried surface (Figs. 1 and 2). The aim of this is to create a barrier between the blood vessel and the erosive pancreatic secretions in case of leak (see Fig. 3).

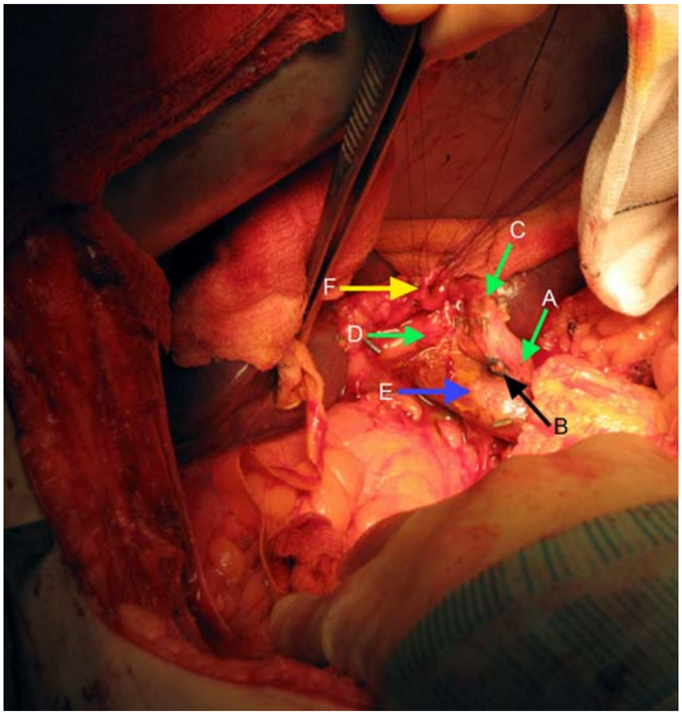

**FIGURE 1.** Relevant anatomical features that can be seen during a Whipple's procedure. A, Common hepatic artery; B, gastroduodenal artery stump; C, left hepatic artery; D, right hepatic artery; E, portal vein; F, hepatic duct.

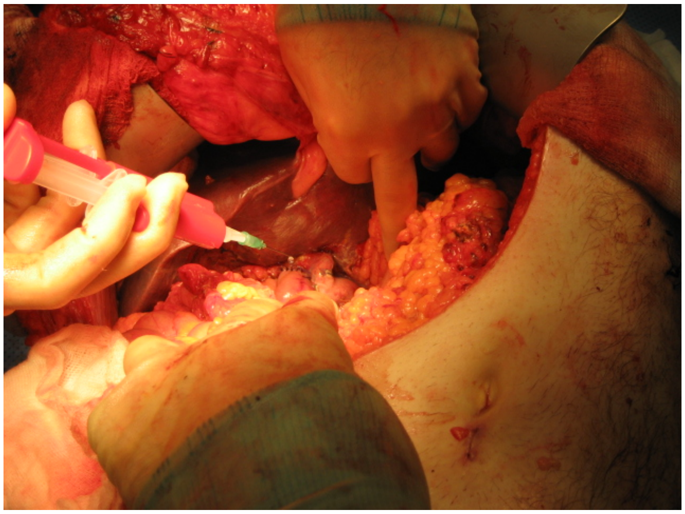

**FIGURE 2.** Application of the hemostatic products via a syringe to the exposed vessels to a thickness of at least 2 mm. Special attention is given to application over the area of the gastroduodenal artery stump.

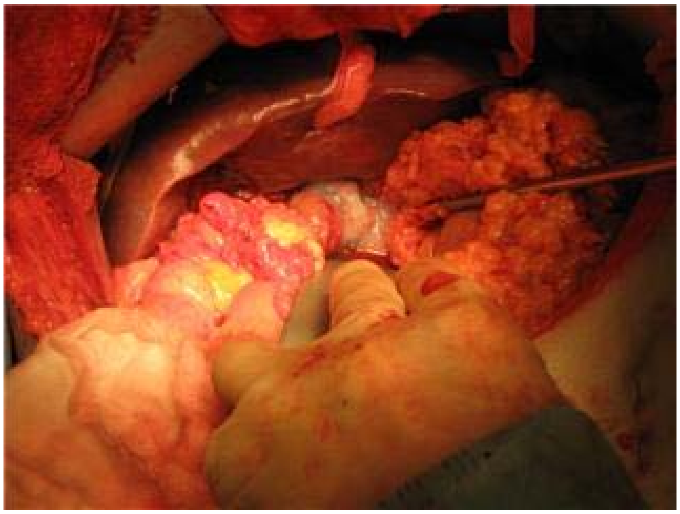

**FIGURE 3.** The final reconstruction can be seen secured with glue.

Occasionally, we have also applied these products to the pancreatic anastomosis as a protective measure; however, we currently do not have clear data to comment on their effectiveness in reducing the incidence of pancreatic leak.

We suggest that this method could be a simple preventive measure to adopt in the daily practice of pancreatic surgeons in order to improve outcomes following PD.
